# Bioreactor mixing efficiency modulates the activity of a prpoS::GFP reporter gene in *E. coli*

**DOI:** 10.1186/1475-2859-8-15

**Published:** 2009-02-25

**Authors:** Frank Delvigne, Mathieu Boxus, Sophie Ingels, Philippe Thonart

**Affiliations:** 1 Fond de la recherche scientifique (FRNS-FRS), Rue d'Egmont 5, 1000 Bruxelles, Belgium; 2 Faculté Universitaire des Sciences Agronomiques, Unité de Bio-industries/CWBI, Passage des Déportés 2, 5030 Gembloux, Belgium; 3 Université de Liège, Service de technologie microbienne/CWBI, Sart-Tilman, B40, 4000 Liège, Belgium; 4 Faculté Universitaire des Sciences Agronomiques, Unité de Biologie cellulaire et moléculaire. Avenue Maréchal Juin, 13, 5030 Gembloux, Belgium

## Abstract

**Background:**

Extensive studies have shown that up-scaling of bioprocesses has a significant impact on the physiology of the microorganisms. Among the factors associated with the fluid dynamics of the bioreactor, concentration gradients induced by loss of the global mixing efficiency associated with the increasing scale is the main phenomena leading to strong physiological modifications at the level of the microbial population. These changes are not fully understood since they involve complex physiological mechanisms. In this work, we intend to investigate, at the single cell level, the expression of the rpoS gene associated with the stress response of *E. coli*. The cultures of the reporter strain have been performed in a small scale reactor as well as in a series of scaled-down bioreactors able to induce extracellular perturbations with increasing level of magnitude.

**Results:**

The rpoS level has been monitored by the aim of a transcriptional reporter gene based on the synthesis of the green fluorescent protein (GFP). It has been observed that the level of GFP increases during the transition from batch to fed-batch phase. After this initial increase, the GFP content of the cell drops, primarily due to the dilution by cell division. However, a significant drop of the GFP content has been observed if using a partitioned bioreactor, for which the mixing conditions are very bad, leading to the exposure of the cells to cyclic and stochastic extracellular fluctuations. If considering the flow cytometric profile of the cell to cell GFP content, this drop has to be attributed to the appearance of segregation at the level of the GFP content among the microbial population.

**Conclusion:**

The generation of extracellular perturbations (in the present case, at the level of the sugar concentration and the dissolved oxygen level) has led to a drop at the level of the rpoS expression level. This drop has to be attributed to a segregation phenomenon in microbial population, with a major sub-population exhibiting a low expression level and a minor sub-population keeping its initial elevated expression level. The intensity of the segregation, as well as its time of appearance during the culture can be related to the bioreactor mixing efficiency.

## Background

Bioreactors are designed in order to promote an optimal environment for growth and/or metabolites synthesis. However, during scale-up, the hydrodynamic efficiency is strongly altered, potentially leading to cell exposure to heterogeneities [1]. Microbial cells are highly sensitive to these heterogeneities and are able to respond by several physiological mechanisms involving a complex set of cellular networks (metabolome, proteome, genome), each exhibiting their own spatial and temporal organization [2]. At this time, several cellular mechanisms associated with the bioreactor heterogeneities remain to be elucidated. These mechanisms can be observed at the lab scale by using the so-called scale-down strategy [3]. The scale-down reactors are able to reproduce at small scale the extracellular fluctuations experienced by the cells in large-scale bioreactors. Extracellular fluctuations perceived by the cells depend on the interrelation between two fluid dynamics related processes, i.e. the global mixing efficiency of the reactor governing the spatial intensity of the concentration gradient field (for an aerobic process, mainly the glucose and the dissolved oxygen level), and the circulation paths followed by cells. To this end, a precise definition of the extracellular environment perceived by the microbial cells requires the superimposition of the two hydrodynamic mechanisms [4,5]. At this level, difficulty comes from the fact that the circulation process is partly governed by random phenomena, giving rise to a stochastic process at the level of the heterogeneities perceived by the cells [5-7]. Several scale-down apparatus have been described in order to reproduce these heterogeneities [3], i.e. controlled reactors [8], two interconnected lab-scale stirred bioreactors [9-11] and lab-scale stirred reactor connected with a tubular section [12,13], all these systems being based on the principle that the microbial cells must be submitted to oscillating or stochastic (partially random) fluctuations to reproduce the flow conditions encountered at large-scale. At the physiological level, the primary impact of the heterogeneities generated by the different scale-down strategies is a drop at the level of the overall biomass yield and this effect has been observed in several studies involving scale-down reactors [12,14,15]. Other physiological parameters that have been followed during scaling down and scaling up studies are : side products excretion [16], cell viability [13,17-19] and mRNA levels [20]. Magnitude of microbial stress is usually characterized by techniques based on membrane integrity [21]. However, system biology has led a lot of informative material that can be used to find new parameters in order to characterize more precisely microbial stress in bioreactors [2]. These progresses have notably allowed to get more insight about regulation mechanisms involved in the stress response of *E. coli*. Microbial cells have developed signal transduction systems to sense environmental state and to induce coordinated expression of genes involved in the appropriate stress response, this response being critical for the microorganism to adapt and survive [22,23]. For bacteria, the coordination of gene expression program is mediated by small proteins, called sigma factors σ, that bind to RNA polymerases (RNAP). Sigma factors increase the affinity of RNAP for specific promoter regions, according to the class of sigma factor. *E. coli *presents seven classes of sigma factors. The main (or housekeeping) σ factor is involved in the transcription from a majority of the promoters. The six other σ factors, called alternate σ factors, induce the activation of more specific promoters involved in the response to specific environmental stimuli (e.g., heat shock, pH shift,...). Among these alternate factors, σ^S^, coded by rpoS, is the master regulator of the general stress response. System biology approaches have allowed to get more insight at the level of the organization of the transcriptional network of *E. coli*, showing that rpoS is among the global regulator located at the top level of the hierarchical structure [24]. The gene rpoS controls the expression of more than 50 genes [25], and its inactivation makes the cells more vulnerable to stress conditions [22]. It is thus interesting to monitor the activity of this gene as a global stress reporter for microbial cells cultivated in heterogeneous reactors. We therefore used a prpoS transcriptional reporter based on the green fluorescent protein expression. Similar construction has been previously used to monitor the effect of various stresses experienced in the environment, such as the osmotic stress [26,27]. A very recent publication involves a nar::gfp reporter to monitor oxygen availability during *E. coli *cultivations in lab scale reactors [28]. In this work, we propose to extent the application of GFP sensor to monitor the effect of several bioprocess specific stresses, i.e. mainly glucose excess, limitation and starvation and oxygen exhaustion.

## Results

### Global trends observed for the different reactor configurations

**Figure 1:**
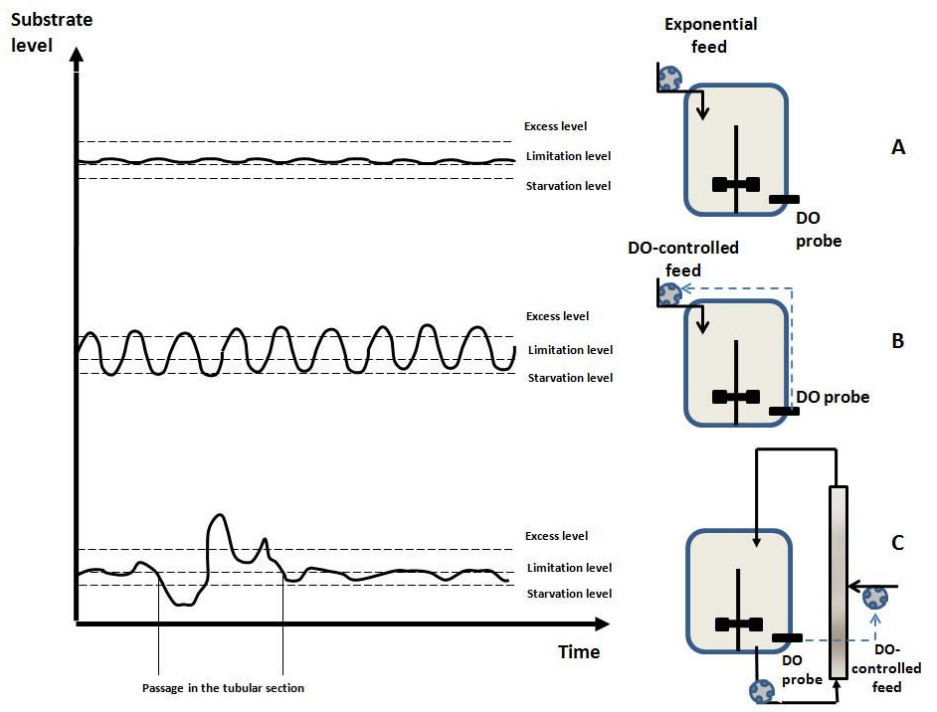
**scheme of the different reactor configuration used to study the influence of the extracellular heterogeneities on *E. coli *and expected substrate profiles**. A : well mixed reactor with exponential feed control ; B : well mixed reactor with an ON/OFF feed control base on the DO level, leading to the appearance of oscillating extracellular conditions ; C : partitioned reactor with an ON/OFF feed control based on the DO level, the glucose being introduced at the level of the middle of the tubular part. The last configuration generate oscillating extracellular conditions for the microbial cells located in the stirred part of the reactor and the same cell can be stochastically exposed to strong concentration gradient in the tubular part.

**Figure 2:**
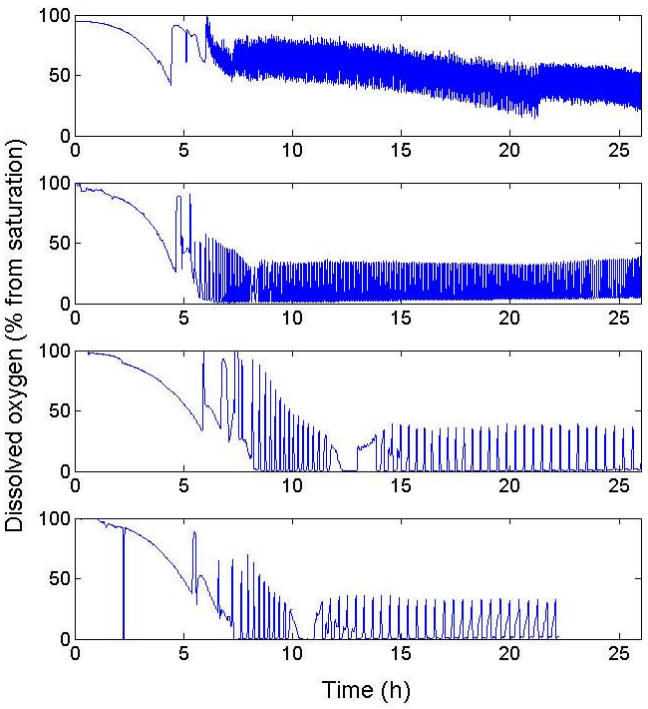
**dissolved oxygen profiles obtained for the different reactor configurations**. From top to bottom: well-mixed reactor exponential feed ; well-mixed reactor ON/OFF DO feed control ; partitioned reactor Q_recirc _= 18 L/h ON/OFF DO feed control ; partitioned reactor Q_recirc _= 36 L/h ON/OFF DO feed control.

**Figure 3:**
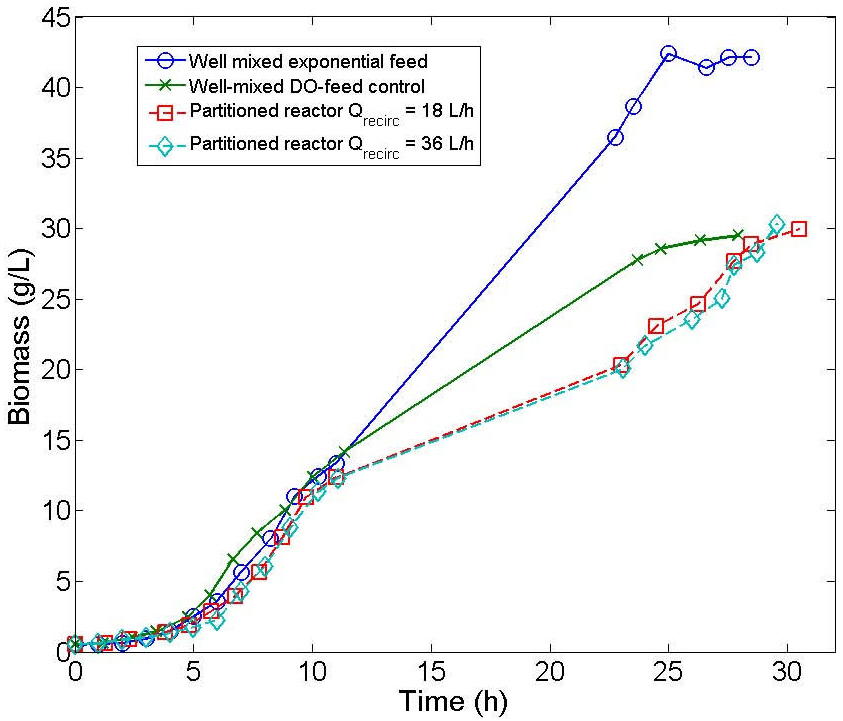
**microbial kinetics for the different bioreactor configurations**.

Culture of *E. coli *is generally performed in fed-batch reactor with precise control of the substrate addition in order to reach high cell densities and to avoid the excretion of by-products [29]. Three cultivation strategies have been considered in order to alter progressively the mixing efficiency of the reactor (figure 1). At first, a reference culture in a well-mixed reactor with an exponential feed has been performed (figure 1A). In a second time, cyclic perturbations at the level of the sugar concentration and dissolved oxygen level have been generated by aim of an abrupt ON/OFF DO-feed control (figure 1B). In a third time, these perturbations have been enhanced by addition of a tubular section to the original apparatus and by performing the DO-feed control at the level of this section (figure 1C). The partitioned reactors used in this study are based on the scale-down configurations (plug-flow reactor PFR connected to a stirred tank reactor STR) used in previous works [12,15,18], except for the DO-controlled feed. In the case of the well mixed bioreactor with exponential feed, hydrodynamics is improved and the resulting extracellular conditions are therefore expected to be the best the growth of the microorganisms. In fact, some limited heterogeneities have to be expected, owing to the pulses generated by the feed pump, but these pulses are rapidly attenuated by the mixing performances of the reactor (typically, about a few seconds for a stirred, small scale, bioreactor). This configuration generates an overall substrate profile slightly above the limitation level (figure 1A). During scale-up, mixing efficiency drops, leading to the development of extracellular heterogeneities. This case study has been reproduced at small scale by using a bioreactor with an ON/OFF dissolved oxygen feed control. Since the regulation trend is not smoothed by a proportional-integral-differential (PID) controller, the glucose is delivered in excess when the dissolved oxygen level rises above 30% from saturation and is subsequently followed by a period of glucose limitation and starvation until the dissolved oxygen level reach again the set point. This situation is depicted at figure 1B, by substrate profile oscillating between the excess and the limitation level. However, the situation is more complex in a large-scale bioreactor, where the extracellular conditions met by the cells do not simply follow an oscillatory trend, and are function of the mixing efficiency of the bioreactor and of the circulation pattern followed by the cells. This latter can be assimilated to a stochastic process [4] and induces a significant amount of noise at the level of the extracellular environment met by the microorganisms. This case figure has been tentatively reproduced at small-scale by the use of a partitioned scale-down, for which glucose is injected at the level of the tubular part (figure 1C). In this case, cells are exposed to limitation and starvation at the level of the stirred bioreactor and are stochastically pumped through the tubular part where they encounter a strong starvation in the first half of the section and a strong glucose excess in the second half. The effects of the different culture conditions at the level of the general bioprocess trend are depicted at figure 2 for the DO level, and at figure 3 for the microbial growth. It can be clearly seen from figure 3 that there is a significant difference at the level of the biomass evolution between the well-mixed reactor with an exponential feed and the perturbed reactors (with the well-mixed DO-controlled reactor exhibiting a slightly higher biomass yield than the partitioned reactors). These results are in accordance with previous scale-down studies [17,18]. In the case of the DO-controlled reactor, the oxygen level fluctuations govern the dynamics of the substrate addition (figure 2). For the well mixed-non partitioned reactor, it can be seen that, for the dissolved oxygen profile, frequency of substrate addition is relatively high. In the case of partitioned bioreactors, more spaced dissolved oxygen peaks can be distinguished, revealing a significantly lower substrate feeding frequency. In this case, glucose is punctually added in large quantities, leading to strong concentration gradient inside the partitioned reactor, and particularly throughout the tubular section. In the case of well mixed reactor performed with an exponential feeding strategy, the dissolved oxygen fluctuations can be attributed to the fact that the feeding algorithm is designed in order to operate close to the substrate and dissolved oxygen uptake capacity of the microorganism.

### Extracellular fluctuations experienced in heterogeneous bioreactors induce segregation of the rpoS expression level

**Figure 4:**
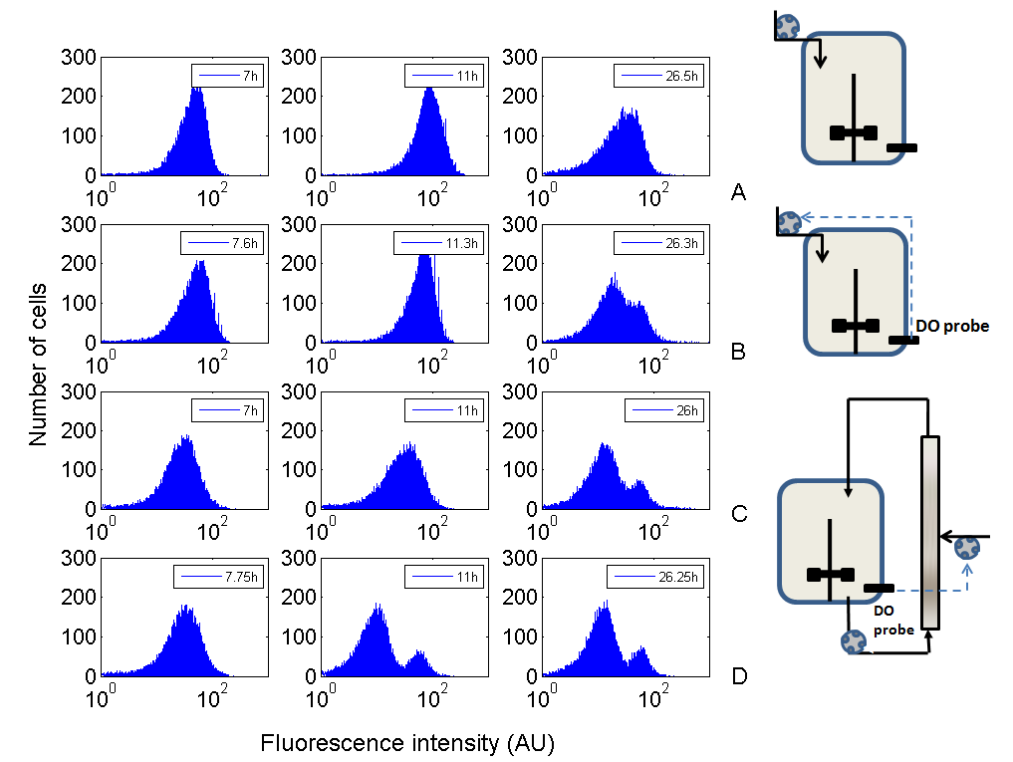
**Evolution of the segregation phenomena in function of the bioreactor mixing efficiency**. From top to bottom, the histograms are presented in decreasing order at the level of the mixing efficiency. A : well-mixed reactor with exponential feed ; B : well-mixed reactor with DO-feed control ; C : partitioned reactor with Q_recirc _= 36 L/h ; D : partitioned reactor with Q_recirc _= 18 L/h. The full set of data can be viewed in additional file 1, 2, 3 and 4.

In addition to the global parameters described in the previous section (biomass and dissolved oxygen evolution), the fate of the microbial population has been monitored by flow cytometry. This technique has been applied for all the bioreactor configurations in order to estimate the expression level associated to a rpoS::GFPmut2 construction inserted in the cells. For each reactor configuration, flow cytometry profiles have been observed at different times during the culture. The whole set of data is too important to be inserted in the text and is thus presented in additional files (additional files 1, 2, 3 and 4, for each reactor configuration). The main information is summarized at figure 4, showing the distribution of rpoS expression level at different times and for each reactor configuration. Segregation at the level of the rpoS expression level can be clearly observed in the case of cultures performed in partitioned reactors. Indeed, two sub-populations of cells can be distinguished, the first sub-population exhibiting a low expression of rpoS and the second one exhibiting a high level of expression associated to the promoter of this gene. The same phenomena can also be observed in a lower extent for the late stages of the culture performed in the well-mixed reactor with an ON/OFF DO-controlled feed. It is thus interesting to note that the intensity as well as the time of appearance of the segregation are both correlated with the mixing efficiency of the reactor. For the less efficient reactor (i.e., the partitioned reactor with the lower recirculation flow rate), the segregation appears early, and two sub-populations are clearly visible after 11 hours of culture. For the partitioned reactor with the higher recirculation flow rate, segregation is only partially observed at 23 hours and begins to be clearly visible at 26 h. For the well-mixed reactor with the DO-feed control, the segregation appears very lately during the culture (26 h) and is fairly attenuated if compared to the previous cases.

**Figure 5:**
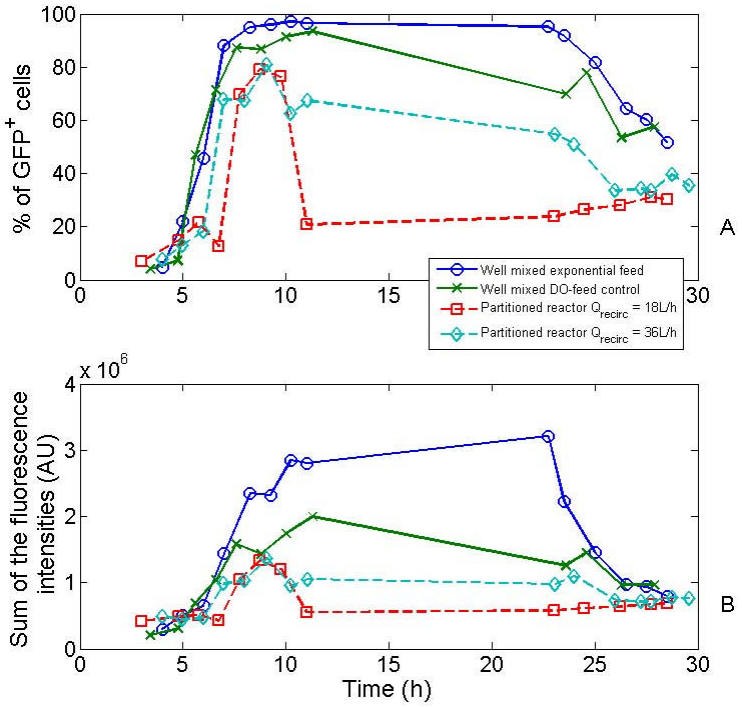
**evolution of the fraction of GFP^+ ^cells and of the total fluorescence over the different cultures**. The GFP+ population has been determined as explained in the Methods section. The total fluorescence has been obtained by summing the raw flow cytometry data collected for 30,000 cells per run (prpoS::GFPmut2 reporter system).

The evolution of the GFP expression is depicted at figure 5, considering evolution of the amount of GFP positive cells and the evolution of the total fluorescence. For all the reactor configurations tested, it can be seen that the GFP is extensively synthesized between 5 and 10 hours of culture, this period including the transition from the batch phase to the fed-batch phase. This transition is generally associated with a decrease of the growth rate, a condition leading to the induction of rpoS [30,31]. In the same context, it has been shown that ppGpp, an alarmone responsible for the induction of rpoS, is strongly synthesized after transition from bath to the fed-batch phase [32]. After this global increase of the GFP content of the cells, fluorescence intensity tends to remain constant for the well-mixed reactor with an exponential feed, and tends to drop in the case of the heterogeneous reactors. It is interesting to note that the drop is significantly higher in the case of the partitioned bioreactors, where the extracellular perturbations experienced by the cells are very intensive. Indeed, after the beginning of the fed-batch regulation, the curves depicted at figure 5 show a progressive decrease proportional to the intensity of the heterogeneities encountered in the corresponding reactor. The drop in the amount of GFP^+ ^cells (or more roughly, the drop of the fluorescence intensity of the microbial population) is enhanced by the segregation mechanism illustrated at figure 4. This effect is distributed among the population since in the worst case, i.e. partitioned bioreactor with the lower recirculation flow rate, there is a minor subpopulation showing a standard level of expression (standard means that in this case, the fluorescence intensities of these cells are in the range of those encountered in the well-mixed reactor). In front of these considerations, it appears that the stochasticity inherent to the extracellular noise (in our case mainly with respect to the glucose and oxygen availability) enhance the segregation phenomena.

**Figure 6:**
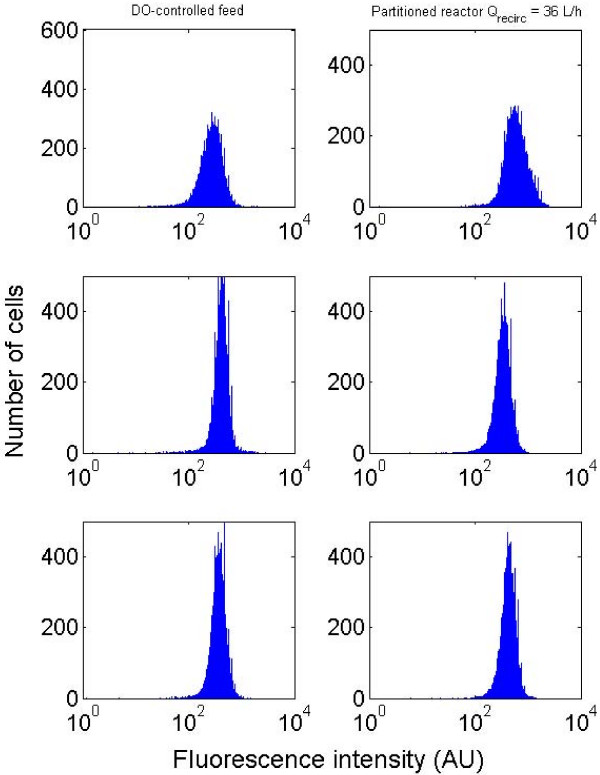
**evolution of the flow cytometry profile (pcyaA:GFPmut2 strain)**. Left : DO-controlled reactor ; right : partitioned reactor with Q_recirc _= 36 L/h. From top to bottom the cultivation time is respectively 6 h, 11 h and 26 h.

However, several artifacts can be linked to GFP expression at the level of the heterogeneous reactors. Indeed, it has been reported that heterogeneities induce several changes at the level of the microorganism physiology (viability, membrane integrity,...) and it is not excluded that these changes have an impact at the level of protein conformation, leading to a fluorescence drop. In order to examine this hypothesis, additional experiments have been carried out with another reporter strain carrying a cyaA::GFPmut2 plasmid. The cyaA codes for adenylate cyclase, a key enzyme promoting the conversion of ATP to cAMP. As shown in figure 6, this enzyme seems to be constitutively expressed, since flow cytometry profiles show a peak of expression of high intensity with a narrow dispersion from cell to cell (the sensitivity of the photomultiplier has been lowered compared with the measurements carried out with the rpoS strain in order to overcome channel saturation as reported in the Methods section). For the experiments carried out with the cyaA reporter strains, the dissolved oxygen profile as well as the microbial growth curve is similar to that obtained in equivalent hydrodynamic conditions with the rpoS reporter strain (data not shown). The most important information depicted at figure 6 is that the evolution of the expression profile is not affected by the bioreactor hydrodynamics (the full datasets are available in additional files 5 and 6, and photographs of pcyaA::gfp cells are available in additional file 7). This result suggests that the rpoS expression level recorded in the previous experiments is not promoted by artifacts linked with the protein maturation and conformation.

### The characteristic time for GFP synthesis does not allow to obtain instantaneous informations about the partitioned reactors hydrodynamics

**Figure 7:**
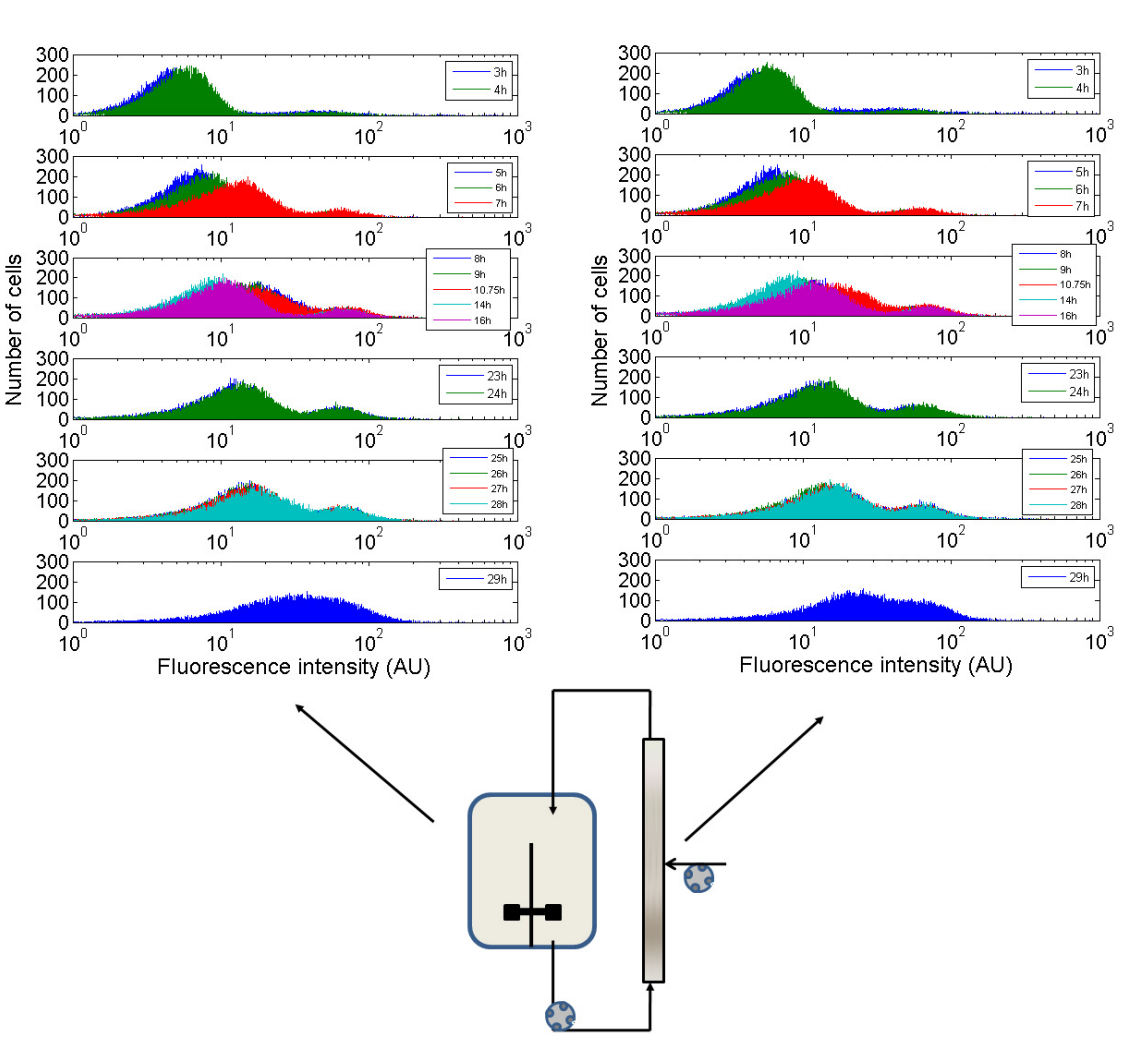
**evolution of the flow cytometry profile (prpoS::GFPmut2 strain) in the two sections of the partitioned bioreactor (Q_recirc _= 18 L/h)**. Rigth panel : tubular section ; left panel : mixed section. GFP intensity profiles have been classified in order to represent the different phases of the culture. From top to bottom : initial state in the batch phase, transition from batch to fed-batch phase, early fed-batch phase, late fed-batch phase, early stationary phase, late stationary phase.

In order to gain some insights about the GFP synthesis process and to assess the reproducibility of the results, the experiment performed in the partitioned reactor with a Q_recirc _= 18 L/h has been reiterated. Microbial growth curve and dissolved oxygen profile are similar to those obtained in the previous experiment with the same reactor configuration (data not shown). Samples for flow cytometry have been taken both at the level of the stirred part and at the outlet of the tubular part. The results are shown at figure 7. The segregation phenomenon is observed again at the early stages of the fed-batch process. No significant differences can be observed at the level of the GFP expression profiles between samples collected at the level of the stirred reactor and the tubular part. This observation implies that the characteristic time related to GFP synthesis and maturation is larger than the characteristic time for the exchanges between the mixed and the tubular part of the partitioned bioreactor. In the working conditions used to run the experiment, the theoretical residence time inside the tubular part of the reactor working at a recirculation flow rate of 18 L/h is 3.33 minutes. Informations obtained in the literature indicate that the GFPmut2 variant is a fast folding protein with a synthesis and maturation time of 4 minutes [33] (the original GFP exhibits a maturation time of approximately 95 minutes [34]). It is thus normal to observe no difference of expression level between the two parts of the reactor. In this case, the GFP expression level represents the stress accumulated by the cells exposed to extracellular fluctuations. In order to point out physiological parameters giving the instantaneous state of the cells, other molecules with a higher synthesis rate, must be considered. The mRNA transcript analysis constitutes in this case a performant tool, since several techniques, such as RT-PCR [10], and more recently an at-line technique based on a sandwich hybridization assay [35], are available. However, mRNA based techniques don't allow to obtain information at the single cell level, but have to be overlooked as a good complement to the reporter gene technology.

### The segregation is reversible and seems to have no significant effect on cell viability

**Figure 8:**
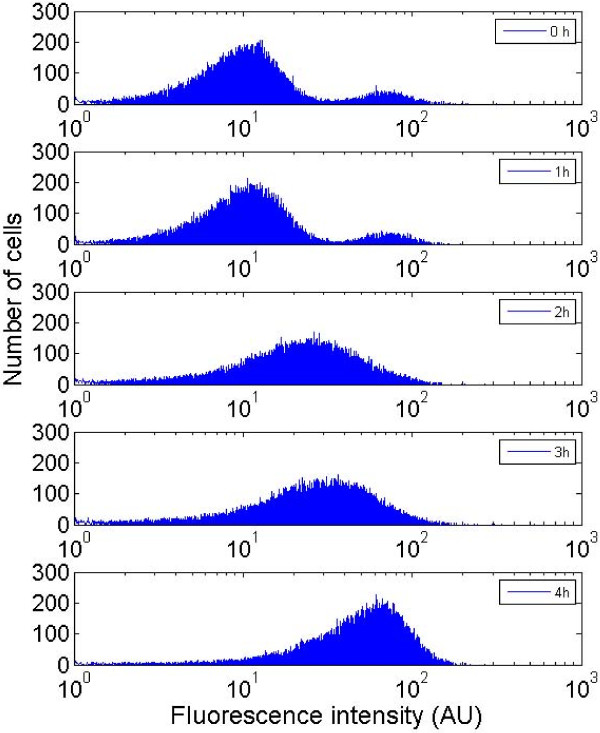
**evolution of the flow cytometry profile (prpoS::GFPmut2 strain) for a fraction of cells collected at the level of a partitioned reactor (Q_recirc _= 18 L/h) and put in fresh medium**. The culture has been performed in shake flask in the same medium used for the culture performed in bioreactor. From top to bottom, the figure depicts the evolution of the flow cytometry profile at different time of the culture.

**Figure 9:**
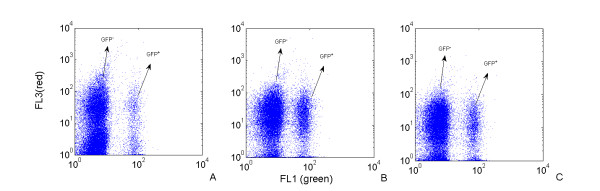
**cell viability at the level of the two subpopulations (GFP^+ ^and GFP^-^)**. The green channel (FL1) depicts the GFP expression level and the red channel (FL3) depicts the degree of PI absorption by the cells with a compromised membrane. Sample taken after 16 h (A), 25 h (B), 30 h (C) of culture in a partitioned reactor (Q_recirc _= 18 L/h).

At the late stage of the stationary phase of culture depicted at figure 7, it appears, by comparison with the expression profiles obtained in the well-mixed reactor, that the microbial population shifts from a bimodal to a standard unimodal expression profile. This result is confirmed if taking a sample at the level of the partitioned reactor and by putting the collected cells in fresh medium. As shown at figure 8, the population is shifted from a bimodal to an unimodal GFP expression profile after 2 hours of culture in a fresh medium. These results tend to indicate that the mechanisms leading to segregation are reversible and disappear when cells are cultivated without extracellular fluctuations. In the same order of idea, the viability of the cells has been checked at the level of the two subpopulations (GFP^+ ^and GFP^-^). As shown at figure 9, no difference can be observed at the level of the PI intensity (FL3 channel) between the GFP^+ ^and the GFP^- ^populations. This suggests that cell viability is not correlated with segregation at the level of the stress gene expression. However, and owing to the fact that a lot of mechanisms are involved in rpoS regulation, more precise informations should be obtained if considering the two sub populations separately (e.g., by using cell sorting techniques).

## Discussion

An *E. coli *reporter strain has been cultivated in different reactor configurations with decreasing level of mixing efficiency. The goal of this work is to observe a physiological parameter that can be easily monitored, i.e. in our case the GFP fluorescence intensity resulting from the activation of the rpoS promoter, in order to put this parameter in relation with the bioreactor mixing efficiency. We have observed a significant decrease of the fluorescence level when mixing conditions were altered.

The following discussions will be mainly focused on possible explanations of the segregation phenomena observed at the level of the prpoS level and to propose some practical applications of the finding pointed out in this work.

When exposed to stress, *E. coli *is able to respond by the coordinated expression of a set of genes [22,36]. In normal, non stress, conditions, bacteria have a housekeeping sigma factor (σ^70^) which governs the main transcriptional machinery. In stress conditions, bacteria have also alternate sigma factors that are able to redirect RNAP to specific promoters. *E. coli *has six alternate sigma factors (i.e., σ^S^, σ^H^, σ^N^, σ^E^, σ^F^, σ^Fecl^) that respond to different cellular stresses [36]. In fluctuating environmental conditions, these alternate sigma factors are in competition with the housekeeping sigma factor. For example, when exposed to nutrient starvation, the σ^S ^coded by the rpoS gene and the σ^70 ^coded by the rpoD gene enter in competition [31,37]. These genomic responses correspond to two distinct strategies developed by the cell to cope with nutrient limitation. In the first case figure, cells react by inducing a set of genes involving the global enhancement for the nutrient uptake in order to lead to an improvement of the growth. This first strategy is usually called the hunger response and is triggered by the housekeeping sigma factor σ^70^. In the second case figure, cells induce a stringent response involving a complete subset of genes that will lead to a stationary, dormant state. This pathway corresponds to the general stress response triggered by the σ^S ^subunit, the product of the rpoS gene that has been tracked throughout this work. When exposed to limitation, there is a constant competition of the two subunits for the core RNA polymerase. In several cases, prolonged starvation leads to the appearance of rpoS mutants lacking in the general stress response. The generally accepted explanation about the appearance of such mutants is an unbalance of the competing process with the prevalence of the σ^70 ^at the level of the core RNA polymerases, resulting in a general reduction of the expression of the promoters associated with the general stress response. Another justification is that rpoS makes *E. coli *less competitive in hunger conditions, since it decreases the expression of high affinity transport pathway [38]. In such conditions, rpoS mutation can be viewed as a selective advantage and has been defined as a specific phenotype (GASP for Growth Advantage in Stationary Phase) [39,40].

However, the picture is by far more complex since stress response in bacteria is governed by complex regulatory networks. The rpoS regulation can be performed at different levels: transcription, mRNA turnover and proteolysis [25]. At the transcriptional level, rpoS is induced by ppGpp, an alarmone synthesized under stress conditions. This alarmone is very important for the modulation of starvation response in fed-batch bioreactors [41,42]. The amount of rpoS transcripts can be regulated by the presence of ppGpp, by but also by mutation at the level of the corresponding synthases (RelA for the synthesis of ppGpp in case of amino acid starvation, and SpoT for the synthesis of ppGpp in case of carbon starvation). However, additional difficulties arise when considering ppGpp as a master regulator of stress response. Indeed, ppGpp modulates competition of σ factors for core RNA polymerase and can thus induce promoters other than σ^S ^dependent [43]. On the other hand, ppGpp is not absolutely required for the activity of σ^S ^dependent promoters, because underproduction of σ^70 ^(housekeeping) or mutation in rpoD restored the expression of σ^S ^dependent promoters [44]. A protein, DksA, acts as a coregulator of genes controlled by ppGpp, and can compensate for the loss of ppGpp [36,45]. Thus, possible mutations accumulating in the genes involved in this regulation can be unnoticed while studying prpoS activity. In front of these considerations, the phenomena observed in this work can be only understood if considering the entire network or sub-network involved in the general stress response, highlighting the need for integrating system biology into biochemical engineering approaches. Indeed, system biology proposes simplified numerical tools to study the complexity of gene network. As an example, the concept of bistability involves gene networks in order to describe the global behavior of a population of cells, characterized by a bimodal distribution for a physiological characteristic among the population [46]. Bistability is induced by noise and several models involving interactions between subset of genes (called network motifs) have been proposed to explain its occurrence [47-49]. More specifically, bistability arises when a master regulator of the gene network is involved. In our case, we have investigated the level of expression of the rpoS gene, which is a recognized master regulator of the *E. coli *genes network belonging to the top level of the hierarchical structure of this network [24]. It is also recognized that bistability (or in our case segregation phenomena) is strongly enhanced when the stochasticity of the extracellular fluctuations is increased [50]. This observation is in accordance with our results, showing that the most intensive segregation phenomena has been observed in the partitioned bioreactors for which the extracellular perturbations are strongly governed by a stochastic mechanism at the level of the passage between the stirred part and the tubular part [6].

A possible explanation of the segregation phenomena can be postulated on the basis of the properties of GFP. As said before, rpoS regulation occurs at three levels: transcription, mRNA stability and proteolysis. Without excluding the numerous phenomena occurring at the transcriptional level, and considering the properties of GFPmut2, proteolysis can be involved in the fluorescence drop associated with scale-down effect. Indeed, GFPmut2 is a fast folding mutant of the original protein with a high stability, and it is generally assumed that the lifetime of this protein is longer than the cell cycle [51]. In our case, segregation phenomena, i.e. strong fluorescence decreases for a major part of the microorganisms (about 80%), occurs within one hour (see flow cytometry profiles in additional files), suggesting that fluorescence drop cannot be only attributed to GFP dilution due to cell division. The possible explanation involves proteolysis of the GFP content of the cells. This phenomenon has not been observed in the case of the pcyaA::gfp reporter strain cultivated in the same conditions. However, in this case, the GFP content of cells was significantly higher than for prpoS strain, and proteolysis can be less marked.

The results acquired during this work suggest a strong modulation of the rpoS promoter in front of the intensity, but also the frequency at which perturbations occur. Two kinds of scale-down reactor have been used throughout the study. The first one is the DO-controlled reactor inducing oscillating extracellular conditions according to the glucose and dissolved oxygen level. In this kind of reactor, all the cells are exposed alternatively to the oscillating conditions. The second one is the partitioned bioreactor which is different from the previous one. In this kind of reactor, cells are exposed to oscillating environmental conditions, but a fraction of cells can be exposed to stochastic fluctuations when crossing the tubular section. It seems that the stochastic component plays an important role by enhancing the intensity of the segregation mechanism among the population. Surprisingly, sub-optimal conditions induce a drop of prpoS activity. This observation can be explained by the induction of stress proteases that has been proposed previously as the possible mechanism leading to the diminution of GFP content of the cells. However, the segregation mechanism cannot be entirely attributed to the stochastic nature of the fluctuations, i.e. to the physical fragmentation of the reacting volume into a well-mixed and a heterogeneous zone, because the segregation mechanism has also been observed in the DO-controlled reactor. Lin *et al*. [52] have reported complex mechanisms having strong influence on protein synthesis by *E. coli *in function of the frequencies of the extracellular segregation. In this case, additional informations can be collected if using a method with a smaller response time than the reporter gene technology. Indeed, it has been reported that the characteristic time related to GFP is too large, even if a fast folding mutant is used and if the characteristic time associated to the exchange process in the partitioned reactors is important, to observe the instantaneous stress level of a cell in a particular flow region. No difference of GFP level has been observed between the mixed and the nonmixed part of the partitioned reactor. This fact highlights the potentialities of mRNA based techniques as a complementary tool for GFP expression studies.

Considering the different rpoS expression levels obtained in function of the hydrodynamic efficiency of the reactor, this gene can be considered as a good reporter of the physiological stress experienced by *E. coli *in process conditions. In practise, some specific probes are available for the on-line monitoring of the GFP fusion proteins and could be used to make some useful control of the bioreactor in direct relation with the physiological status of the cells (the initial concept of fluorescence probe that can be fit in bioreactors has been developed by Randers-Eichhorn and co-workers [53], and this concept has been more recently improved [54]). The main limitation with this approach lies on the fact that these fluorescence probes only give a signal averaging the physiological status over the whole population. However, it has been shown that there is a strong segregation of the physiological status of the cells in the heterogeneous reactors, leading to a subpopulation with a strong expression level and another one with a low expression level. In a bioreactor control perspective, it will be thus very useful to adapt an on-line flow cytometer. The efficiency of such automated apparatus have been estimated in the literature but is not yet routinely used for the on-line monitoring of bioreactors [55].

## Conclusion

Previous scale-down experiments involve the observation of direct (mRNA analysis, cellular viability) or indirect (global growth rate, oxygen consumption rate,...) physiological parameters, but it is the first time that a GFP reporter strain is used in this context. The use of a prpoS::GFPmut2 reporter strain shows an increasing level of segregation when the mixing efficiency of the bioreactor is altered. Experiments carried out in different perturbed reactor (i.e., where glucose and dissolved oxygen fluctuations occur) show that a fraction of the cellular population lose its stress response capabilities in heterogeneous conditions. It appears thus that the intensity, as well as the frequency at which extracellular perturbations occur, are of importance in the repartition of gene expression level among the population. This imply that prpoS::gfp reporter is thus a promising tool for monitoring stress level encountered by microbial cells in process conditions.

## Methods

### Strain and cultivation media

E. coli K12 MG1655 bearing a pMS201 (4260 bp) plasmid with either the prpoS::GFPmut2 or the pcyaA::GFPmut2 transcriptional reporter and a kanamycin resistance gene. These strains comes from a cloning vector library elaborated at the Weizmann Institute of Science [33]. The strain is maintained at -80°C in working seeds vials (2 mL) in solution with LB media and with 40% of glycerol. The precultures and cultures have been performed on a defined liquid medium containing (in g/L): K_2_HPO_4 _14.6, NaH_2_PO_4_.2H_2_O 3.6; Na_2_SO_4 _2; (NH_4_)_2_SO_4 _2.47, NH_4_Cl 0.5, (NH_4_)_2_-H-citrate 1, glucose 5, thiamine 0.01, kanamycin 0.1. Thiamine and kanamycin are sterilized by filtration (0.2 μm). The medium is supplemented with 3 mL/L of trace solution, 3 mL/L of a FeCl_3_.6H_2_O solution (16.7 g/L), 3 mL/L of an EDTA solution (20.1 g/L) and 2 mL/L of a MgSO_4 _solution (120 g/L). The trace solution contains (in g/L) : CoCl_2_.H_2_O 0.74, ZnSO_4_.7H_2_O 0.18, MnSO_4_.H_2_O 0.1, CuSO_4_.5H_2_O, CoSO_4_.7H_2_O. The precultures are performed in 600 mL of the above mentioned medium in shake flask at 37°C. The bioreactor experiments are performed initially with 10 L of the above mentioned medium. The feed solution contains 2 L of the basal medium but with all the concentrations doubled, except for the glucose which is at 500 g/L. During the culture, cell growth has been monitored by optical density at a wavelength of 600 nm. Cell dry weight has been determined on the basis of filtered samples (0.45 μm) dried during 24 hours at 105°C.

### Reactor configurations

Three kinds of reactor configuration have been used and are schematized at figure 1. The stirred vessel is a standard bioreactor (vessel diameter: 0.22 m; initial working volume: 10 L) equipped with four equally spaced baffles and a single rushton disk turbine with 6 blades. Agitation rate is maintained at 500 min^-1 ^throughout the culture. Air is injected through a ring sparger. Air flow rate is maintained at 10 L/min during the batch phase and is increased to 40 L/min during the fed-batch phase. During the fed-batch phase the reactor internal pressure is increased to 0.4 bars in order to improve oxygen solubility. In the case of the partitioned reactors, stirred reactor is connected to a tubular section having a diameter of 0.05 m and a length of 1 m. The tubular section contains 14 static mixing elements (Kenics) in order to keep a plug-flow hydrodynamics. Recirculation between the stirred vessel and the tubular section is ensured by a peristaltic pump (Watson Marlow series 323). For each assay, temperature is maintained at 37°C and at pH 7 by a digital control system (ABB). The pH is maintained constant by the addition of ammonia solution (25%). Dissolved oxygen is measured by a polarographic probe (Mettler Toledo). In the case of the exponential feed, the equation has been calculated and adjusted based on a previous fed-batch experiment. The equation has been implemented in a MatLab program driving the feed pump (Watson Marlow series 101 U/R) working by pulse on the basis of a flow rate of 15 mL/min. In the case of the DO-controlled feed, the pump activation is ensured by the ON/OFF contact option at the level of the DO transmitter (Knick oxy 2402) with a set point of 30% from saturation. In the case of well-mixed reactors, glucose is added by a special port at the top of the vessel. In the case of the partitioned reactors, glucose is added at the mid length of the tubular part, inducing the appearance of a glucose limited zone above the injection port and a glucose excess zone below the injection port.

### Flow cytometry analysis

The analysis of the GFP expression level has been performed with a FACscan (Becton Dickinson) flow cytometer. Samples are taken directly from the reactor and are diluted in 900 μL of PBS and 100 μL of a cycloheximide solution (1 mg/mL) in order to stop protein synthesis. For each measurement, 30,000 cells are analyzed (GFP is excited at 488 nm and emission signals are collected by using filters at 530 nm). The measurements are repeated 3 times at different FL1 channel intensities (FL1 at 480, 550 and 620 for the rpoS strain and FL1 at 400, 480 and 550 for the cyaA strain). The results have been analyzed by the CellQuest (Becton Dickinson) software and are subsequently exported to WinMDI and MatLab for further analysis. The GFP negative fraction of cells has been determined on the basis of the initial FL1 distribution for each reactor experiment. The initial FL1 distribution is very comparable from a reactor condition to another and corresponds to a very low GFP emission (monitored by epifluorescence microscopy). Cell viability estimation is carried out by using propidium iodide (PI) at a working concentration of 5 μg/ml. Before addition of PI, cells are washed with PBS. Sample is then divided in two parts, the first being untreated and the second being stained with PI for 5 minutes at room temperature.

## Competing interests

The authors declare that they have no competing interests.

## Authors' contributions

FD planned the experiments, performed the first half of the experiments carried out in bioreactors and wrote the final version of the manuscript. MB performed the flow cytometry experiments. SI performed the second half of the experiments carried out in bioreactors. PT supervised the work. All authors read and approved the final version of the manuscript.

## Supplementary Material

Additional File 1 **Evolution of the flow cytometry GFP profile (prpoS::GFPmut2) during the culture performed in a bioreactor with an exponential feed control**. Histograms have been classified in order to represent the different phases of the culture. From top to bottom : initial state in the batch phase, transition from batch to fed-batch phase, early fed-batch phase, late fed-batch phase, stationary phase. Click here for file

Additional File 2 **Evolution of the flow cytometry GFP profile (prpoS::GFPmut2) during the culture performed in a bioreactor with a DO-controlled feed**. Histograms have been classified in order to represent the different phases of the culture. From top to bottom : initial state in the batch phase, transition from batch to fed-batch phase, early fed-batch phase, late fed-batch phase, stationary phase. Click here for file

Additional File 3 **Evolution of the flow cytometry GFP profile (prpoS::GFPmut2) during the culture performed in a partitioned bioreactor with a Q_recirc _= 36 L/h**. Histograms have been classified in order to represent the different phases of the culture. From top to bottom : initial state in the batch phase, transition from batch to fed-batch phase, early fed-batch phase, late fed-batch phase, stationary phase. Click here for file

Additional File 4 **Evolution of the flow cytometry GFP profile (prpoS::GFPmut2) during the culture performed in a partitioned bioreactor with a Q_recirc _= 18 L/h**. Histograms have been classified in order to represent the different phases of the culture. From top to bottom : initial state in the batch phase, transition from batch to fed-batch phase, early fed-batch phase, late fed-batch phase, stationary phase. Click here for file

Additional File 5 **Evolution of the flow cytometry GFP profile (pcyaA::GFPmut2) during the culture performed in a well-mixed reactor with a DO-controlled feed**. Histograms have been classified in order to represent the different phases of the culture. From top to bottom : initial state in the batch phase, transition from batch to fed-batch phase, early fed-batch phase, late fed-batch phase, stationary phase. Click here for file

Additional File 6 **Evolution of the flow cytometry GFP profile (pcyaA::GFPmut2) during the culture performed in a partitioned bioreactor with a Q_recirc _= 36 L/h**. Histograms have been classified in order to represent the different phases of the culture. From top to bottom : initial state in the batch phase, transition from batch to fed-batch phase, early fed-batch phase, late fed-batch phase, stationary phase. Click here for file

Additional File 7 ***E. coli *pcyaA::GFPmut2 clone observed under fluorescence microscopy (well-mixed reactor with a DO-feed control)**. Photographs taken by epifluorescence microscopy at 23 hours of culture. Click here for file
