# Supplementary figures and images for: Bioreactor mixing efficiency modulates the activity of a prpoS::GFP reporter gene in E. coli
Source: Microb Cell Fact. 2009 Feb 25;8:15. doi: 10.1186/1475-2859-8-15 (PMC2650683; doi:10.1186/1475-2859-8-15)

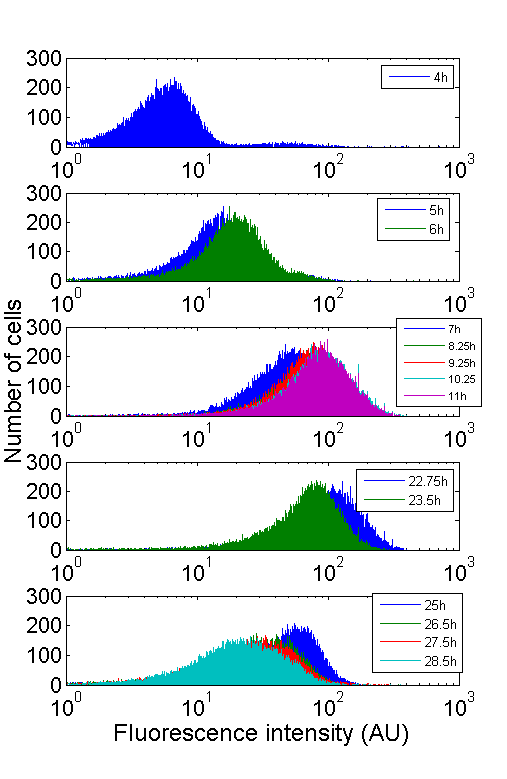

Supplement: Additional File 1 — Evolution of the flow cytometry GFP profile (prpoS::GFPmut2) during the culture performed in a bioreactor with an exponential feed control. Histograms have been classified in order to represent the different phases of the culture. From top to bottom : initial state in the batch phase, transition from batch to fed-batch phase, early fed-batch phase, late fed-batch phase, stationary phase. [file 1475-2859-8-15-S1.tiff]

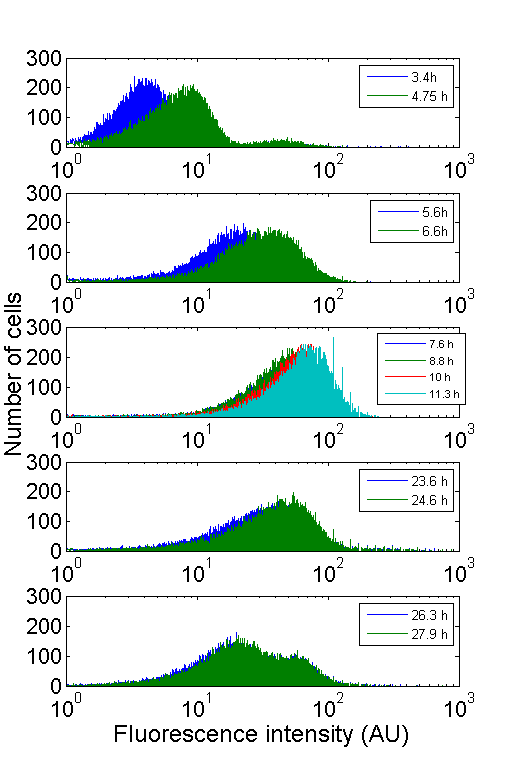

Supplement: Additional File 2 — Evolution of the flow cytometry GFP profile (prpoS::GFPmut2) during the culture performed in a bioreactor with a DO-controlled feed. Histograms have been classified in order to represent the different phases of the culture. From top to bottom : initial state in the batch phase, transition from batch to fed-batch phase, early fed-batch phase, late fed-batch phase, stationary phase. [file 1475-2859-8-15-S2.tiff]

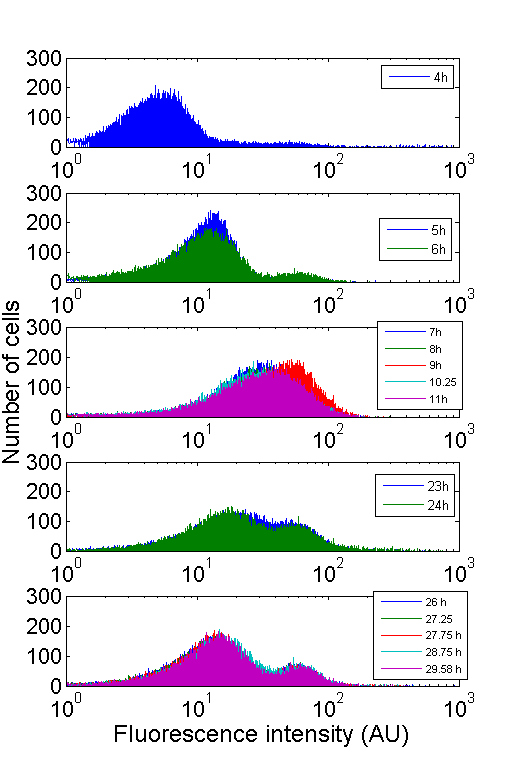

Supplement: Additional File 3 — Evolution of the flow cytometry GFP profile (prpoS::GFPmut2) during the culture performed in a partitioned bioreactor with a Qrecirc = 36 L/h. Histograms have been classified in order to represent the different phases of the culture. From top to bottom : initial state in the batch phase, transition from batch to fed-batch phase, early fed-batch phase, late fed-batch phase, stationary phase. [file 1475-2859-8-15-S3.tiff]

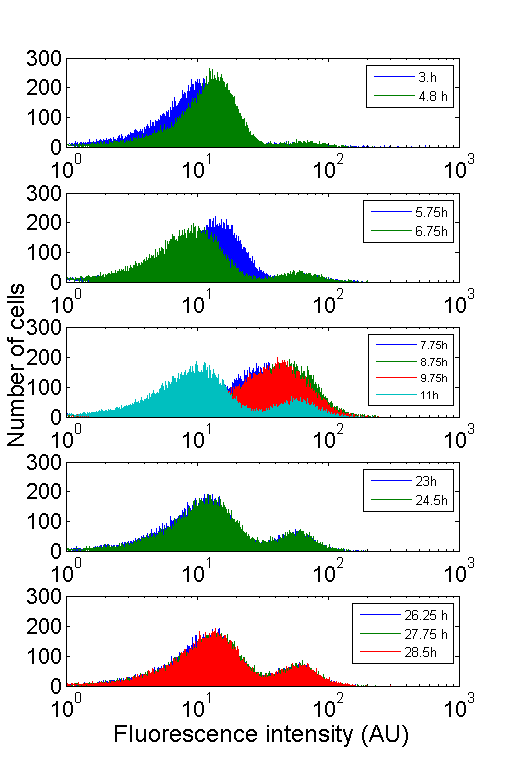

Supplement: Additional File 4 — Evolution of the flow cytometry GFP profile (prpoS::GFPmut2) during the culture performed in a partitioned bioreactor with a Qrecirc = 18 L/h. Histograms have been classified in order to represent the different phases of the culture. From top to bottom : initial state in the batch phase, transition from batch to fed-batch phase, early fed-batch phase, late fed-batch phase, stationary phase. [file 1475-2859-8-15-S4.tiff]

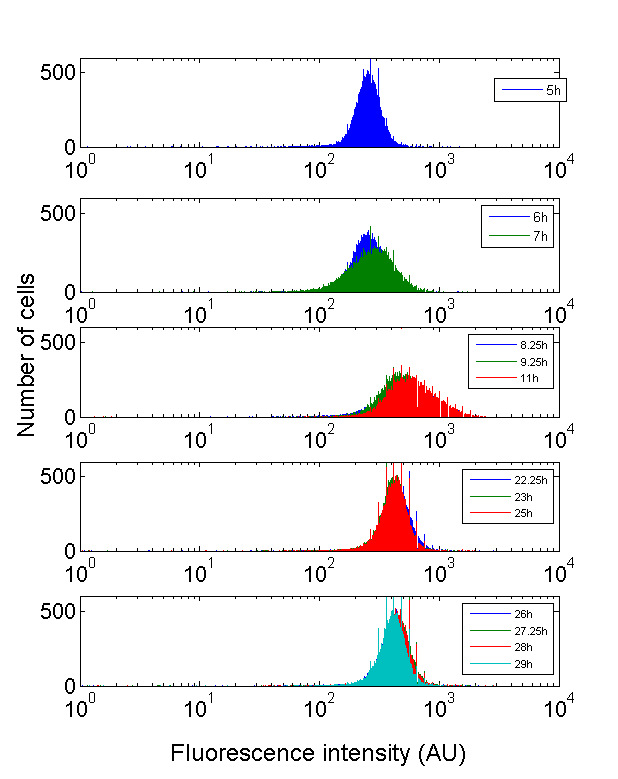

Supplement: Additional File 5 — Evolution of the flow cytometry GFP profile (pcyaA::GFPmut2) during the culture performed in a well-mixed reactor with a DO-controlled feed. Histograms have been classified in order to represent the different phases of the culture. From top to bottom : initial state in the batch phase, transition from batch to fed-batch phase, early fed-batch phase, late fed-batch phase, stationary phase. [file 1475-2859-8-15-S5.tiff]

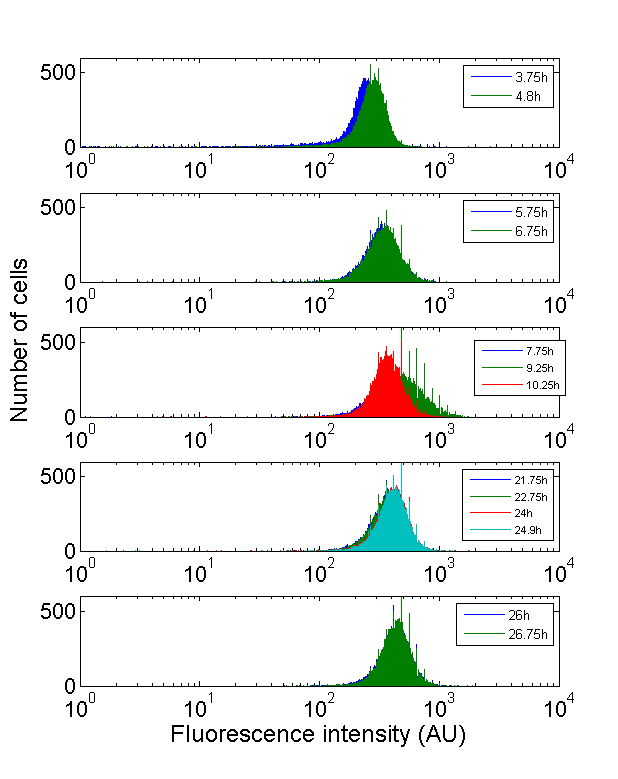

Supplement: Additional File 6 — Evolution of the flow cytometry GFP profile (pcyaA::GFPmut2) during the culture performed in a partitioned bioreactor with a Qrecirc = 36 L/h. Histograms have been classified in order to represent the different phases of the culture. From top to bottom : initial state in the batch phase, transition from batch to fed-batch phase, early fed-batch phase, late fed-batch phase, stationary phase. [file 1475-2859-8-15-S6.tiff]

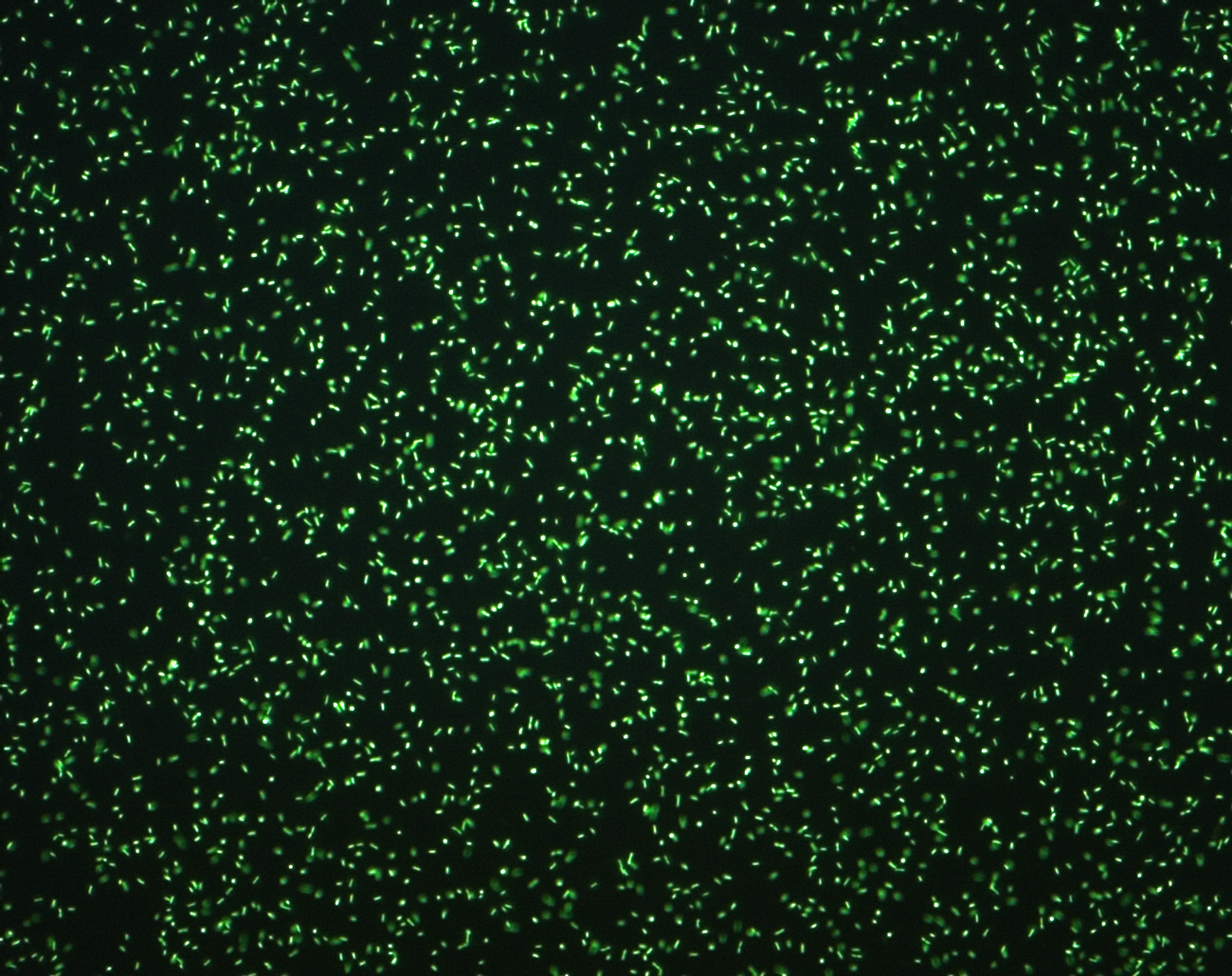

Supplement: Additional File 7 — E. coli pcyaA::GFPmut2 clone observed under fluorescence microscopy (well-mixed reactor with a DO-feed control). Photographs taken by epifluorescence microscopy at 23 hours of culture. [file 1475-2859-8-15-S7.tiff]
